# Using 3 Health Surveys to Compare Multilevel Models for Small Area Estimation for Chronic Diseases and Health Behaviors

**DOI:** 10.5888/pcd15.180313

**Published:** 2018-11-01

**Authors:** Yan Wang, James B. Holt, Fang Xu, Xingyou Zhang, Daniel P. Dooley, Hua Lu, Janet B. Croft

**Affiliations:** 1 Division of Population Health, National Center for Chronic Disease Prevention and Health Promotion, Centers for Disease Control and Prevention, Atlanta, Georgia; 2 Economic Research Service, US Department of Agriculture, Washington, DC; 3 Boston Public Health Commission, Boston, Massachusetts

## Abstract

**Background:**

We used a multilevel regression and poststratification approach to generate estimates of health-related outcomes using Behavioral Risk Factor Surveillance System 2013 (BRFSS) data for the 500 US cities. We conducted an empirical study to investigate whether the approach is robust using different health surveys.

**Methods:**

We constructed a multilevel logistic model with individual-level age, sex, and race/ethnicity as predictors (Model I), and sequentially added educational attainment (Model II) and area-level poverty (Model III) for 5 health-related outcomes using the nationwide BRFSS, the Massachusetts BRFSS 2013 (a state subset of nationwide BRFSS), and the Boston BRFSS 2010/2013 (an independent survey), respectively. We applied each model to the Boston population (2010 Census) to predict each outcome in Boston and compared each with corresponding Boston BRFSS direct estimates.

**Results:**

Using Model I for the nationwide BRFSS, estimates of diabetes, high blood pressure, physical inactivity, and binge drinking fell within the 95% confidence interval of corresponding Boston BRFSS direct estimates. Adding educational attainment and county-level poverty (Models II and III) further improved their accuracy, particularly for current smoking (the model-based estimate was 15.2% by Model I and 18.1% by Model II). The estimates based on state BRFSS and Boston BRFSS models were similar to those based on the nationwide BRFSS, but area-level poverty did not improve the estimates significantly.

**Conclusion:**

The estimates of health-related outcomes were similar using different health surveys. Model specification could vary by surveys with different geographic coverage.

## Introduction

Public health data for small geographic areas have been in demand by local health authorities in recent years to help them with public health prevention program planning, resource allocation, health policy formulation, and health care decision-making and delivery. Local level health data typically are not available through national or nationwide health surveys, such as the National Health Interview Survey and Behavioral Risk Factor Surveillance System (BRFSS). Consequently, small area estimation (SAE) methods are applied to meet this need. SAE methods can be broadly divided into design-based methods (estimates evaluated based on design-based distribution) and model-based methods (estimates rely solely on the model specified); both include model construction. The developments in both methods are available (1–4).

Multilevel regression and poststratification (MRP) is a model-based SAE approach that was first used in estimating state-level preferences from national polls. It was developed by Gelman and Little (5) and was later extended (6–8). It includes multilevel modeling using individual survey responses from national polls to generate estimates by demographic–geographic subgroups via poststratification. It was evaluated and validated by several studies in state-level public opinion estimation and was suggested to produce accurate estimates using simple models (6,7,9). We applied the MRP framework to health data and developed a more flexible approach to obtain estimates for health-related measures at any target small area level (10), and it has been validated at both county (11) and city levels (12). We then applied it to the nationwide BRFSS data to generate estimates of US city- and census tract–level prevalence for a select set of chronic disease and health behavior measures related to public health priorities and impact. This collaboration, called the 500 Cities Project, is ongoing between the Centers for Disease Control and Prevention (CDC), the CDC Foundation, and the Robert Wood Johnson Foundation. So far, the data have been widely used by state or local health departments, community services, and academic researchers for multiple purposes. We have received feedback from the users that there are growing interests and needs from local health departments to use their own data, such as state BRFSS or local health surveys, to obtain SAE for chronic diseases and health behaviors. These local departments may not be able to obtain the geocoded national or nationwide surveys, or they may be interested in different health-related outcomes that are not available from national or nationwide surveys; they also tend to develop their own technical capacity in SAE.

There are challenges to meeting such needs. First, few local health surveys collect population health data annually because of a lack of regular funding, and local health surveys usually have limited geographic coverage and a small set of health-related outcomes. Second, it is not certain whether the statistical model used with the national or nationwide health survey data for SAE is appropriate for use with local health surveys, even for the same health-related outcomes. Third, the statistical model is affected by many explanatory variables, so an optimal model that includes a set of explanatory variables is needed to obtain better prediction. Traditional model fit statistics, such as Akaike information criterion, Bayesian information criterion (13), and Bayesian Deviance Information criteria (14,15) are used to evaluate model fitness for the data and may not be appropriate for model prediction in SAE. Increasing more explanatory variables may make a model fit the data better but may also have a risk of overfitting, which introduces bias in small area estimation (13). It is unclear how the model specification varies with different data sources and health-related outcomes.

Our objective was to assess if this approach was appropriate for SAE with state BRFSS and local health surveys and how model specification varied by different data sources and health-related outcomes. To do this, we needed a local health survey and a state BRFSS, respectively, to generate the model-based estimates of select health measures for the same city. We also would compare these estimates with the benchmark and with the nationwide BRFSS model-based estimates. It is not easy to find “true” values for the health measures; therefore, the direct survey estimates from the local survey usually serve as the benchmark for accuracy evaluation, and ideally the local survey had similar designs and questions with the data source of SAE. The Boston BRFSS was the available independent local health survey that we had to meet the objective, so we used it and the Massachusetts BRFSS to construct a series of multilevel models to estimate 5 health-related outcomes (diabetes, high blood pressure, physical inactivity, binge drinking, and current smoking) for Boston, Massachusetts. We also compared them with the Boston BRFSS direct estimates and the nationwide BRFSS model-based estimates for evaluation.

## Methods

**Data sources and health-related outcomes.** The nationwide BRFSS is a state-based, annual, random-digit–dialed landline and cellular telephone survey representative of the noninstitutionalized adult population aged 18 years or older residing in the 50 states, the District of Columbia, and US territories. In the BRFSS 2013, data from 483,865 respondents residing in 3,136 counties from 50 states and the District of Columbia were collected. From these data, we extracted the Massachusetts state BRFSS data with 15,071 respondents from 14 counties. The Boston BRFSS was a separate survey, which is administered by the Boston Public Health Commission and that focused on the health of residents in Boston. It was conducted in 2010 and 2013 and included many of the same BRFSS core questions, as well as questions particular to Boston. Boston BRFSS in both years featured a nonoverlapping, dual frame of both landline and cellular telephones that had a random-digit–dialing sample design. We combined Boston BRFSS 2010 and 2013 data for a total of 7,340 respondents from 29 zip codes. We selected 5 health-related outcomes (diagnosed diabetes, diagnosed high blood pressure, physical inactivity, current smoking, and binge drinking), which were assessed in all 3 surveys. All outcomes were categorized as binary variables (1 = yes and 0 = no) for the 3 surveys. The definition and categorization is available in the 2013 BRFSS code book (www.cdc.gov/brfss/annual_data/2014/pdf/codebook14_llcp.pdf).

**Models and procedures.** We used the MRP approach (12) to generate model-based estimates of each outcome using 3 data sources. First, we constructed a multilevel logistic model for each outcome using the nationwide BRFSS, which included age (aged 18 to ≥80 y), sex (male, female), race/ethnicity (non-Hispanic white, non-Hispanic black, American Indian or Alaska Native, Asian, Native Hawaiian or other Pacific Islander, other race, two or more races, and Hispanic), and state-nested county random effects (Model I). We then constructed 2 sequential models that added 4 categories of educational attainment (Model II) and then the county-level percentage of adults below 150% of the poverty threshold (Model III). County-level poverty data for Model III were obtained from the American Community Survey 2009–2013 (https://factfinder.census.gov/faces/nav/jsf/pages/index.xhtml). 

We also modeled Massachusetts BRFSS data in the same way with the same predictors in the model except that the county-level random effect was not state-nested. Finally, we constructed multilevel models using Boston BRFSS data. Predictors were the same, but race/ethnicity and educational attainment were categorized differently, given that the distributions in Boston BRFSS data were different from Massachusetts BRFSS and nationwide BRFSS data (Table 1). For the Boston BRFSS, we condensed race/ethnicity to 5 categories (non-Hispanic white, non-Hispanic black, Asian/Native Hawaiian/other Pacific Islander, other race, and Hispanic) and educational attainment to 2 categories (less than a bachelor’s degree and bachelor’s degree or higher). Each respondent had zip code identification, so we included zip code–level random effect in the multilevel models.

**Table 1:** Weighted Distribution of Demographic Characteristics and Health Related Outcomes in 3 Surveys, BRFSS 2013, Massachusetts BRFSS 2013, and Boston BRFSS 2010/2013^a^

Predictor	Boston BRFSS^b^ (n = 7,340)	Massachusetts BRFSS (n = 15,071)	BRFSS (n = 483,865)
**Age, y**			
18–24	18.7	13.4	13.0
25–29	13.6	7.9	8.1
30–34	12.9	8.4	9.1
35–39	7.5	6.9	7.7
40–44	8.2	9.0	9.0
45–49	7.3	8.4	8.0
50–54	7.3	10.3	10.0
55–59	6.0	8.8	8.5
60–64	6.0	8.0	8.0
65–69	3.9	6.0	6.0
70–74	3.0	4.3	4.5
75–79	2.5	3.7	3.7
≥80	3.2	4.9	4.3
**Sex**			
Male	47.2	47.8	48.6
Female	52.8	52.2	51.4
**Race/ethnicity**			
Non-Hispanic white	50.3	76.8	65.1
Non-Hispanic black	21.8	5.8	11.8
Non-Hispanic American Indian or Alaska Native	0.0	0.5	1.1
Non-Hispanic Asian	2.3	5.3	4.7
Non-Hispanic Native Hawaiian or other Pacific Islander	0.0	0.3	0.2
Non-Hispanic other	9.5^b^	1.0	0.4
Non-Hispanic two or more races	0.0	1.2	1.4
Hispanic	16.1	9.1	15.4
**Educational attainment**			
Less than grade 12	14.3	11.4	8.4
Grade 12 or GED	20.0	26.4	29.2
Some college	24.1	27.0	27.5
College or higher	41.6	35.1	34.9
**<150% Poverty level, median % (IQR)^c^**	27.8 (19.9–38.0)	17.5 (13.4–20.2)	24.7 (18.8–29.4)
Diabetes	7.9	8.5	10.0
High blood pressure	24.3	29.4	32.4
Physical inactivity	22.5	23.5	26.3
Current smoking	18.7	16.6	18.2
Binge drinking	25.5	19.4	16.5

Abbreviations: BRFSS, Behavioral Risk Factor Surveillance System; GED, general educational diploma; IQR, interquartile range.

a Values are percentages unless otherwise indicated.

b The percentage in the Boston BRFSS includes non-Hispanic Native Hawaiians, non-Hispanic other Pacific Islanders, and other non-Hispanic races only.

c The median percentage of poverty <150% is the median among zip codes in the Boston BRFSS, the median among counties in the Massachusetts BRFSS, and the median among counties in the nationwide BRFSS, respectively.

We used SAS version 9.3 (SAS Institute, Inc) to implement the multilevel logistic models. We applied the default residual pseudo-likelihood estimation method to estimate the model parameters and selected variance components as the model’s covariance structure. We applied the predicted probabilities based on the fitted multilevel logistic models to the Boston population counts by age, sex, and race/ethnicity (2010 Census data), and we used the poststratification to obtain the prevalence estimates of each health outcome at the city level in Boston. We used Monte Carlo simulation with 1,000 replicates of model parameters to obtain the mean model-based estimates and their 95% confidence intervals (CIs). We evaluated the accuracy of the model-based estimate for each outcome by determining the absolute difference between the model-based estimate and the Boston BRFSS direct estimate and assessing whether the model-based estimate fell within the 95% CI of the Boston BRFSS direct estimate. The Boston BRFSS direct estimates were calculated using SUDAAN (RTI International), accounting for complex survey design. Statistical significance was set at *P* < .05.

## Results

Compared with the Massachusetts BRFSS and the nationwide BRFSS, the Boston BRFSS had younger respondents, more non-Hispanic black and Hispanic respondents, and more highly educated respondents (Table 1). The distribution of characteristics was similar in Massachusetts BRFSS and in the nationwide BRFSS.

Table 2 shows the Boston BRFSS direct estimates and the model-based estimates of the 5 health outcomes using the 3 surveys. In Model I of the nationwide BRFSS, estimates of diabetes, high blood pressure, physical inactivity, and binge drinking fell within the 95% CI of the corresponding Boston BRFSS direct estimate. However, Model I underestimated current smoking; the BRFSS model-based estimate was 15.2%, and the Boston BRFSS direct estimate was 18.7%. Adding educational attainment in Model II and county-level poverty in Model III provided point estimates that were closer to Boston BRFSS direct estimates, particularly for current smoking (15.2% in Model I compared with 18.5% in Model III). It also suggests that these 5 predictors are adequate for estimation of the selected health-related outcomes. Model I of the Massachusetts BRFSS produced estimates that fell within the 95% CI of the corresponding Boston BRFSS direct estimate for all outcomes except physical inactivity and current smoking (Table 2). The model-based estimate of current smoking increased from 15.5% in Model I to 18.7% in Model II with the addition of educational attainment. Educational attainment in the Massachusetts BRFSS was not significantly associated with binge drinking in Model II, hence the estimate of binge drinking was unchanged (24.4% in both Models I and II). County-level poverty (Model III) was not significantly associated with any of the 5 outcomes in the Massachusetts BRFSS multilevel models (*P* < .05), and adding it only slightly improved the accuracy of binge drinking estimate. For the other 4 outcomes, adding county-level poverty to Massachusetts BRFSS models either did not change the estimation or slightly overestimated the prevalence in comparison with Model II (current smoking).

**Table 2:** Model-Based Estimates of Prevalence of Selected Health Outcomes, by BRFSS Survey Data Source, BRFSS 2013, Massachusetts BRFSS 2013, and Boston BRFSS 2010/2013

Data Source	Diabetes	High Blood Pressure	Physical Inactivity	Current Smoking	Binge Drinking
	Percentage (95% Confidence Interval)				
**Boston BRFSS direct estimate**	7.9 (7.2–8.7)	24.3 (23.0–25.7)	22.5 (20.7–24.3)	18.7 (17.3–20.3)	25.5 (23.6–27.2)
**BRFSS model-based estimate**					
Model I: age, sex, race/ethnicity	7.3 (7.2–7.3)	23.7 (23.7–23.7)	20.7 (20.7–20.7)	15.2 (15.2–15.2)	24.3 (24.3–24.4)
Model II: age, sex, race/ethnicity, educational attainment	7.5 (7.4–7.5)	24.5 (24.4–24.6)	22.6 (22.4–22.8)	18.1 (18.1–18.2)	24.2 (24.2–24.3)
Model III: age, sex, race/ethnicity, educational attainment, county-level poverty^a^	7.7 (7.7–7.8)	24.7 (24.5–24.8)	22.8 (22.6–22.9)	18.5 (18.4–18.7)	23.8 (23.7–23.9)
**Massachusetts BRFSS model-based estimate**					
Model I: age, sex, race/ethnicity	7.7 (7.6–7.8)	23.6 (23.5–23.7)	20.3 (20.2–20.4)	15.5 (15.5–15.6)	24.4 (24.3–24.5)
Model II: age, sex, race/ethnicity, educational attainment^b^	8.1 (8.0–8.2)	24.6 (24.4–24.7)	22.4 (22.2–22.6)	18.7 (18.3–19.0)	24.4 (24.3–24.5)^b^
Model III: age, sex, race/ethnicity, educational attainment, county-level poverty^b^^,^ c	8.2 (8.2–8.3)	24.6 (24.5–24.8)	22.4 (22.2–22.7)	19.5 (19.1–19.9)	25.6 (25.4–25.8)^b^
**Boston BRFSS model-based estimate**					
Model I: age, sex, race/ethnicity	7.8 (7.8–7.8)	21.5 (21.5–21.5)	19.5 (19.5–19.5)	15.9 (15.8–15.9)	25.2 (25.2–25.2)
Model II: age, sex, race/ethnicity, educational attainment^d^	8.4 (8.0–8.8)	23.1 (22.7–23.9)	21.6 (21.1–22.2)	18.5 (17.7–19.3)	25.8 (25.4–26.2)
Model III: age, sex, race/ethnicity, educational attainment, zip code–level poverty^d^^,^ e	8.5 (8.1–8.8)	23.1 (22.8–23.5)	21.9 (21.3–22.5)	18.9 (18.0–19.8)	25.9 (25.3–26.4)

Abbreviation: BRFSS, Behavioral Risk Factor Surveillance System.

a All variables were significantly associated with all 5 outcomes of Model III in the BRFSS.

b Educational attainment was not significantly associated with binge drinking in Models II and III in the Massachusetts BRFSS.

c County-level poverty was not significantly associated with any of the 5 health outcomes in Model III of the Massachusetts BRFSS.

d Educational attainment was not significantly associated with binge drinking in Models II and III of the Boston BRFSS.

e Zip code–level poverty was significantly associated with diabetes, physical inactivity, and current smoking, but not significantly associated with high blood pressure and binge drinking in Model III of the Boston BRFSS.

In Boston BRFSS models (Table 2), Model I yielded estimates accurately only for diabetes (model-based estimate, 7.8% vs Boston BRFSS direct estimate, 7.9%) and binge drinking (model-based estimate, 25.2% vs Boston BRFSS direct estimate, 25.5%). Model I underestimated high blood pressure (model-based estimate, 21.5% vs Boston BRFSS direct estimate, 24.3%), physical inactivity (model-based estimate, 19.5% vs Boston BRFSS direct estimate, 22.5%), and current smoking (model-based estimate, 15.9% vs Boston BRFSS direct estimate, 18.7%). Adding educational attainment (Model II) improved the accuracy of estimates of high blood pressure, physical inactivity, and current smoking. For example, the model-based estimate of physical inactivity increased from 19.5% to 21.6%, and the model-based estimate of current smoking increased from 15.9% to 18.5%. Compared with Model II, adding zip code–level poverty (Model III) only slightly changed the model-based estimates in Model II.

## Discussion

We found that with the nationwide BRFSS data, predictors of age, sex, and race/ethnicity yielded accurate model-based estimates of diabetes, high blood pressure, physical inactivity, and binge drinking. Adding educational attainment and county-level poverty improved the accuracy of prediction, particularly for current smoking. The estimates were well-predicted by age, sex, and race/ethnicity, and educational attainment using Massachusetts BRFSS data; county-level poverty was not a strong predictor. Age, sex, and race/ethnicity alone in Boston BRFSS Model I accurately estimated the prevalence of diabetes and binge drinking but not high blood pressure, physical inactivity, or current smoking. Addition of educational attainment improved the accuracy of the estimates.

Current SAE practice in public health could benefit from our findings. We found that age, sex, and race/ethnicity, educational attainment, and area-level poverty were able to explain most of the variations for the 5 selected health related outcomes and were adequate to provide acceptable SAEs. State and local health departments can apply the MRP approach to their BRFSS data for SAE if they are unable to access geocoded national or nationwide surveys or they are interested in different outcomes that are not available from national or nationwide surveys. However, it is notable that the multilevel models must be specified by data source with different geographic coverage, given that geographic contexts play an important role in the relationships between the health-related outcomes and the demographic characteristics (16,17). For example, county-level poverty contributed significantly to the variations in data of the health-related outcomes for the nationwide BRFSS, but it did not improve the models and SAE with Massachusetts BRFSS and Boston BRFSS data. There may be several explanations for this. First, the Massachusetts Mandated Health Insurance Law, implemented in 2006, could have lessened the effect of poverty on health that had been observed in other states. Second, we used area-level percentages of the poverty threshold in the Massachusetts and Boston BRFSS models rather than an individual-level poverty indicator. Third, educational attainment improved the models and SAE with all 3 BRFSS surveys; however, the Boston BRFSS had smaller variation in race/ethnicity and a more highly educated population than the Massachusetts and the nationwide BRFSS. We therefore categorized race/ethnicity and educational attainment differently in the Boston BRFSS models. Fourth, because missing explanatory variables in the multilevel models could be partially compensated for by the random effects (13), we included a county-level random effect with nationwide BRFSS and Massachusetts BRFSS data and a zip code–level random effect with Boston BRFSS data. However, if the data can be geocoded to smaller geographic contexts, such as census tracts, researchers may reconsider the model specification. Finally, model fitting varied by different health-related outcomes. Some chronic diseases, such as diabetes and high blood pressure, and certain health behaviors, such as binge drinking, were highly affected by age; thus, age explained most of the variations among the counties. This finding may explain why Model I accurately predicted the prevalence of diabetes, high blood pressure, and binge drinking. In terms of current smoking, educational attainment likely also played a role. However, educational attainment was not a significant independent predictor for binge drinking estimation. Different health-related outcomes may require different explanatory variables in the multilevel models.

Our findings suggest that the application of the MRP approach to Massachusetts BRFSS and Boston BRFSS with modest sample sizes obtained similar results to the large scale nationwide surveys. Thus, state health departments could produce SAE using their state BRFSS surveys for health-related outcomes of interest for small geographic areas or subpopulation groups that CDC’s 500 Cities Project did not cover. Local health departments will need to address issues unique to their needs when applying the MRP approach for SAE (Table 3).

**Table 3:** Key Questions and Answers for the MRP Methodology, BRFSS 2013, Massachusetts BRFSS 2013, and Boston BRFSS 2010/2013

Questions	Answers
Where can I find additional information on the methodology used in small area estimation?	A summary of small area estimation and the MRP approach can be found in references 5 and 10 in this article.
What surveys can be used for the approach?	State BRFSS or other local health surveys with hierarchical structure and spatial identifier.
Can the approach be used to generate estimates for other areas, such as rural areas?	Yes. The approach can be used to generate estimates for any target small geographic area.
Can the models be used to evaluate the effectiveness of the local public health interventions?	The estimates are generated based on the multilevel models, which include covariates obtained from the source survey. Unless the survey provides such information on local interventions, the model is not able to predict intervention effectiveness.
Can the model be used to track the changes at the local level over time?	The methods in this study are not designed for assessing trends.
Has the methodology been evaluated for accuracy?	The model was evaluated in comparison with direct estimates from local health survey at the county and city levels. Please refer to correlation results in the findings.
Where can I find additional information about the methodology application?	Please refer to the website www.cdc.gov/500cities. For common questions and answers, please refer to https://www.cdc.gov/500cities/faqs/index.htm. For specific questions, please contact 500cities@cdc.gov.

Abbreviation: MRP, multilevel regression and poststratification.

These model-based estimates can be used for their public health program planning and health care decision-making, to compare 2 or more small areas for the purpose of resource allocation, or to compare different subpopulation groups to see if they are equally exposed to a disease or health-related behavior. Specialized local health surveys with spatial identifiers also can be applied as the source data using the MRP framework for the SAE for the local health departments. However, multilevel model specification varies by data structure, geographic coverage, and health-related outcome. 
